# Attitudes Toward and Preconditions for Digital Game–Based Learning Among Public Health and Health Sciences Students: Cross-Sectional Online Survey Study

**DOI:** 10.2196/83347

**Published:** 2026-09-10

**Authors:** Kamil J Wrona, Joanna Albrecht, Anne Krümmel, Leona Aschentrup, Christoph Dockweiler, Timothy Mc Call

**Affiliations:** 1 School of Public Health, Bielefeld University, Universitätsstraße 25, Bielefeld, 33615, Germany, +49 521 106 67873; 2 Department of Social Sciences, Faculty of Arts and Humanities, University of Siegen, Siegen, Germany; 3 School of Public Health, Bielefeld University, Universitätsstraße 25, Bielefeld, 33615, Germany; 4 School of Public Health, Medical School OWL, Bielefeld University, Bielefeld, Germany

**Keywords:** serious games, digital game–based learning, education, survey, public health, health sciences

## Abstract

**Background:**

Digital game–based learning (DGBL) is gaining traction in medical and nursing education, but its integration into public health and health sciences curricula remains limited. This study addresses this research gap by exploring student perspectives, use patterns, and expectations regarding DGBL within German public health study programs.

**Objective:**

The study investigated how public health and health sciences students perceive DGBL and identified conditions that promote or hinder its integration into academic training for public health professionals.

**Methods:**

A cross-sectional online survey was conducted from January 2021 to March 2024. A total of 444 students participated, with 326 completing the questionnaire. The instrument combined closed- and open-ended questions on sociodemographic characteristics, digital media use, attitudes toward DGBL, and structural and pedagogical requirements. Quantitative data were analyzed descriptively using SPSS; qualitative data were evaluated using MAXQDA and Kuckartz structured content analysis.

**Results:**

The sample consisted predominantly of full-time students enrolled in bachelor’s or master’s programs in public health and health communication. Digital media are widely used for communication (n=320, 98.2%), information search (n=291, 89.3%), and entertainment (n=268, 82.2%) in daily student routines. In contrast, video gaming and online shopping play a minor role. Students expressed a generally positive attitude toward DGBL, particularly in practical learning contexts such as exam preparation, visualizing complex content, and knowledge checks. High agreement was found for using DGBL in modules such as Prevention and Health Promotion (n=277, 84.9%), Epidemiology and Statistics (n=271, 83.2%), and Population Medicine (n=251, 76.7%). Preferred devices include desktop PCs, tablets, and smartphones, while immersive technologies such as virtual reality or augmented reality glasses or consoles are less favored. Students highlighted specific prerequisites for DGBL integration: a functioning technical infrastructure (n=321, 98.5%), media-literate teaching staff (n=311, 95.4%), and compliance with data protection standards (n=303, 93%). Furthermore, students valued user-friendly design (n=255, 78.2%), technical reliability (n=299, 91.7%), clear feedback (n=236, 72.4%), and flexibility regarding the time and place of use (n=307, 94.2%). Game elements such as animations, multimedia, and reward systems were appreciated, whereas features such as 3D graphics, avatars, and multiplayer modes were considered less important.

**Conclusions:**

The findings show a high willingness among students to engage with DGBL, especially when its design emphasizes clarity, usability, and relevance to course content. Emotional safety, flexible access, and pedagogical integration are more decisive than immersive game features. To effectively implement DGBL in public health education, developers and educators should cocreate low-threshold, didactically sound applications that align with students’ preferences and study realities. Future research should assess learning outcomes and emotional effects longitudinally and explore scalable implementation strategies.

## Introduction

### Background

The teaching and training of public health professionals face challenges resulting from globalization, demographic and epidemiological changes, and increasing socially induced health inequalities. To develop solutions that serve the interests of populations, it is crucial for research and practice that students acquire the necessary qualifications to develop interdisciplinary approaches to policy issues [1]. It is challenging to train students at the Bachelor’s, Master’s, and doctoral levels to acquire comparable, internationally standardized skills that meet the diverse requirements of the labor market [2]. Digital teaching and learning methods can support the acquisition of skills in this regard and have also been introduced into university didactics at German universities [3-5]. Digital teaching and learning methods may include the use of PowerPoint, online learning platforms with lecture recordings, or online-based exercises and examinations [6]. Digital formats such as serious games or multimedia tools, in which students use online videos to create and learn their own explanatory models, are also already being used in teaching practice [7]. However, few concepts have yet been used within Public Health or Health Sciences programs, although the advantages of e-learning have been clearly identified [8].

### Academic Training for Public Health Professionals

Today, public health is understood by the World Health Organization (WHO) as the science and professional practice needed to prevent disease, prolong life, and promote psychosocial and physical health [9]. It is seen not only as a theory but also as a scientific discipline that ideally operates in all policy areas (“health in all policies”) [10] and forms the basis for the practice of population-based health policy [1]. German universities, as well as universities of applied sciences and arts, impart public health knowledge to academically qualified professionals in bachelor’s, master’s, and doctoral programs. Public health education aims to train experts to contribute to planning and decision-making processes in health care and health-related policy fields; qualify them to develop, implement, and evaluate health prevention and promotion measures; and prepare them for research and teaching in Public Health [11].

To help public health professionals develop the knowledge, skills, and abilities to act appropriately to achieve set goals, the Association of Schools of Public Health in the European Region (ASPHER) lists competencies related to working with individuals and groups as well as organizations and systems. They range from methods and population health to policy, economics, and management. Prospective public health professionals are expected to generate new knowledge in the field of public health using a range of scientific methods, to develop the capacity for critical reflection, and to review and evaluate existing knowledge from different disciplines. They need to be able to distinguish between scientific and practical knowledge as a basis for application- and needs-oriented public health research and practice. In addition, they should develop broad, interdisciplinary thinking and working skills in the context of lifelong learning, particularly in view of increasingly frequent job changes. Therefore, interdisciplinarity is not only a core element of public health for solving public health problems but also a central challenge for professional development in Germany [12].

On the basis of the development of public health with regard to constantly changing environments and target groups, Blättner and Dierks [2] formulate 4 central challenges for public health teaching: medical vs population competence in public health; views vs perspectives of public health students; individual vs population medicine and public health ethics; and national vs international public health, including the role of the state in public health.

### Digital Game Applications in the Field of Health

Play and learning are evolutionarily anchored in human development and are closely linked [13]. In the context of international higher education, the training of medical professionals (eg, physicians and nurses) has already begun to use sophisticated e-learning applications under the designation of *digital serious games* and *digital game–based learning* (DGBL) [3].

DGBL has been defined by Prensky [14] as “[...] any learning game on a computer or online” and thus refers exclusively to digital games. The definition emphasizes the learning process in the context of the game. The user should perceive themselves as learning through a high level of engagement, predominantly as if they were playing [14]. Serious games in research and practice currently refer to digital applications such as computer or video games, although there is no clear consensus on the definition [15]. However, it is clear that the focus is on education rather than entertainment [15,16]. Tolks and Lampert [17] have developed the following criteria for serious games: the aim of the game is to convey content that is not primarily for entertainment purposes, the game must contain pedagogical aspects, and these pedagogical structures must be subordinate to the entertaining factors.

The design and development of serious games for the education of health professionals are also very diverse, and only a few publications report on the (software) development process used for the games [18]. In the context of higher education, serious games and DGBL have already been successfully used in various study programs [4,19-21]. For example, Peng [22] found a positive effect of an educational serious game in the area of healthy eating in the public health subfield of health communication and prevention. In this context, an increase in nutrition and weight-related knowledge, an intention to eat healthier, and greater self-efficacy were achieved. In the field of nursing, Chang et al [23] reported that nursing students who participated in an integrated DGBL program showed significantly higher learning performance, self-efficacy, willingness to learn, and learning satisfaction than those in a control group. As some suitable software already exists in the training of medical personnel, the use of such gaming software for public health professionals is also conceivable. There is also considerable overlap between the 2 professions regarding the acquisition of competencies, although there are only a few serious games available for these target groups.

Several reviews document the effectiveness of various serious gaming elements in (academic) health care education [24-27]. An initial review emphasizes that games can increase learning enjoyment and improve knowledge retention in the long term, although the evidence is limited in some cases [27]. More recent studies show that serious games are at least as effective as traditional learning methods and can improve knowledge, skills, and educational satisfaction [26]. Tori et al [24] also show that gamification elements such as scoring systems and level advancement achieve positive learning effects, particularly in physiotherapy, psychology, and physical education. Another review examined adaptive e-learning elements and found that their effectiveness is inconsistent. However, the adaptation process appears to be more beneficial for the acquisition of skills than for the acquisition of pure factual knowledge [25].

Although numerous studies have demonstrated the effectiveness of serious games in higher education within the health sector, research on the specific attitudes of public health students toward the use of DGBL in public health study programs or health sciences remains limited. There is a lack of empirical evidence on which applications are perceived as beneficial and which factors influence their acceptance and implementation. The aim of the present study is to provide a deeper understanding of health sciences students’ attitudes toward DGBL in public health teaching as well as its possible applications. For this purpose, the application possibilities, performance, and effort expectations as well as the facilitating and inhibiting conditions for the implementation of digital games in public health education at universities will be explicated. Ultimately, the results of the study will allow conclusions to be drawn about possible uses and the design of serious games to promote their implementation and acceptance in public health study programs or health sciences. The following research questions will be explored:

- What are the attitudes of students toward the use of DGBL in public health study programs or health sciences?
- What are the advantages of using DGBL in public health study programs or health sciences?
- What factors promote and inhibit the use of DGBL in public health study programs or health sciences from the perspective of students?

## Methods

### Overview

To obtain an assessment of the possible future use of DGBL in public health study programs or health sciences and to structure the associated attitudes and needs of students, a structured web-based survey was conducted. The cross-sectional online survey was developed during the winter semester of 2020-2021 as part of a learning module of the Master of Public Health program at Bielefeld University and administered via the Unipark (Tivian XI GmbH) survey platform.

### Ethical Considerations

Ethics approval to conduct the study was obtained from Bielefeld University. The ethics committee of Bielefeld University reviewed the research project in accordance with the ethical guidelines of the German Psychological Society and the Professional Association of German Psychologists and found the study to be ethically unobjectionable. The application has been filed under the number 2023-243.

### Study Design and Setting

The questionnaire comprised 22 main questions (or statements) with 130 subquestions and required approximately 20 to 30 minutes to complete. It consisted of closed- and open-ended questions divided into 7 content units, including information on sociodemographic data, course of study, current use of digital media, and students’ affinity for technology. Students’ affinity for technology was assessed using the validated *Technikaffinität–Elektronische Geräte* (TA-EG; Affinity for Technology–Electronic Gadgets) questionnaire [28], which includes the subscales of enthusiasm for technology, perceived competence in handling technology, trust in technological systems, and technology-related fears. Internal consistency of the subscales ranged from acceptable to good (Cronbach α). Enthusiasm for technology showed good internal consistency (α=0.8), while perceived competence in handling technology (α=0.74), trust in technological systems (α=0.65), and technology-related fears (α=0.69) showed acceptable internal consistency. In addition, the questionnaire included self-developed items on attitudes toward DGBL, students’ personal assessment of digital media use during their studies, motivation, and social influences on opinion formation and acceptance. The DGBL items were based on a standardized point-and-click adventure game scenario developed for the Department of Epidemiology.

Although the TA-EG scales [28] are a validated instrument, all additional items were developed specifically for this study to capture attitudes and behaviors related to DGBL in the target population. These items were reviewed by experts in public health education to ensure content validity and clarity.

The DGBL scenario and survey items were subsequently pilot-tested with a small group of 5 students from different study programs. Participants completed the survey and provided qualitative feedback in a structured debriefing session focusing on clarity, wording, and technical implementation. On the basis of this feedback, minor revisions were made to improve comprehensibility and usability. Following the pilot phase, a pretest was conducted with 24 students at Bielefeld University during a course session. The pretest focused on feasibility aspects, including completion time, clarity of items, and technical functionality. As the study primarily relied on previously validated instruments, no formal statistical analyses of reliability or validity were conducted at this stage. Instead, the pretest served to evaluate the overall feasibility of the study procedures.

Participants completed the full survey under real study conditions. Subsequently, feasibility aspects and questionnaire structure were discussed, and minor wording adjustments were implemented based on participant feedback. In addition, technical and content-related aspects of the scenario were refined based on pilot testing to ensure clarity and usability across different devices and study contexts.

The DGBL scenario was implemented as a standardized point-and-click adventure. All participants were provided with the same online version, including identical instructions, navigation options, and interactive elements. The scenario was designed to reflect realistic decision-making situations relevant to public health practice.

### Recruitment and Data Collection

To include different student cohorts, the survey was extended across several semesters. The survey was conducted from January 2021 to March 2024 to include students from multiple semesters and study years across the participating health-related programs. This extended period allowed the study to capture attitudes and experiences from both earlier- and later-stage students, ensuring representation of different cohorts within the programs. The survey was administered in German via an online platform targeting students in health-related programs, including public health, Prevention and Health Promotion, and Medicine, regardless of study level (bachelor’s or master’s). Participants were recruited using an open, convenience sampling approach: invitations were sent via university email distribution lists. For Fulda University of Applied Sciences, one of the authors has a teaching assignment and coordinated access to the student distribution lists in collaboration with the university administration. In addition, the survey was promoted in relevant social media groups on Facebook (Meta Platforms Inc), LinkedIn (Microsoft Corp), Xing (New Work SE), and WhatsApp (Meta Platforms Inc). Three reminders were sent to the institutions enlisted in Textbox 1 to reach out to previously contacted cohorts as well as newly enrolled cohorts in public health or health sciences programs.

Eligibility criteria were minimal. Given the exploratory nature of the study, no formal sample size calculation was conducted.

Textbox 1. **Overview of universities for the recruitment of the sample.**

**Bielefeld University**

- Bachelor of Science
- Master of Science
- Doctoral program
- Bachelor of Arts
- Master of Arts
- Postgraduate studies
- Guest students

**Siegen University**

- Bachelor of Science
- Master of Science

**Fulda University of Applied Sciences**

- Bachelor of Science
- Master of Science

**Bielefeld University of Applied Sciences and Arts**

- Bachelor of Science
- Master of Science
- Bachelor of Arts
- Master of Arts

### Statistical Analysis

The data were imported from the Unipark survey platform into the respective analysis software and processed. To structure the analysis and evaluation of the data, variables were checked for their suitability for the corresponding questions, assigned, and coded accordingly.

For the answers to closed questions, the responses of all participants who fully completed the survey were considered. SPSS (version 30; IBM Corp) software was used for the descriptive statistical analysis (frequency and location parameter analyses) of the dataset. For open-ended questions, all participants who provided an answer were included, and the responses were translated into English. The responses were then content analyzed according to Rädiker and Kuckartz [29] using MAXQDA (VERBI Software) analysis software.

## Results

### Sample Description and Distribution of Characteristics

A total of 326 students from health-related programs completed the questionnaire. The majority were female (n=277), and the largest age group was 18 to 25 years (n=123). Most participants were enrolled in public health (n=114) or health communication (n=98) programs, studied full time (n=298), and were pursuing either a bachelor’s degree (n=198) or a master’s degree (n=121). Furthermore, the median study duration was 4 semesters (Table 1).

**Table 1.** Demographic data of the sample (N=326).

Demographic characteristics	Participants, n (%)
Gender^a^	
Female	277 (85)
Male	42 (12.9)
Diverse	3 (0.9)
Not specified	2 (0.6)
Age (years)^b^	
18-25	123 (37.7)
26-30	33 (10.1)
31-35	14 (4.3)
36-40	4 (1.2)
>40	7 (2.1)
Degree program	
Public Health	114 (35)
Health Communication	98 (30.1)
Health Sciences	35 (10.7)
Health Promotion	32 (9.8)
Health	11 (3.4)
Health Education	10 (3.1)
Health Promotion and Management	6 (1.8)
Applied Health Sciences	5 (1.5)
International Health	5 (1.5)
Public Health Nutrition	4 (1.2)
Health Technology	2 (0.6)
Digital Biomedical and Health Science	1 (0.3)
Health and Nursing Sciences	1 (0.3)
Physiotherapy	1 (0.3)
Dietetics	1 (0.3)
Type of program	
Bachelor	198 (60.7)
Master	121 (37.1)
Doctoral program or free doctorate	3 (0.9)
Postgraduate studies or second degree program	2 (0.6)
Guest auditor	1 (0.3)
Not specified	1 (0.3)
Organizational form of the study program	
Full-time program	298 (91.4)
Part-time program	26 (8)
Doctoral program	1 (0.3)
Visiting program	1 (0.3)
Semester	
1	22 (6.7)
2	71 (21.8)
3	27 (8.3)
4	70 (21.5)
5	35 (10.7)
6	46 (14.1)
>6	55 (16.9)

a Data on “gender” were available for 324 participants; percentages for individual categories are based on the total sample (N=326).

b Data on “age” were available for 181 participants; percentages for individual categories are based on the total sample (N=326).

Overall affinity for technology (TA-EG score) was moderate (median 2.95, SD 0.38). Trust in technology showed the highest mean score (median 3.6, SD 0.56), followed by fear-related attitudes (mean 3, SD 0.44) and competence in using technology (median 2.75, SD 0.44). Surprisingly, enthusiasm had the lowest values among the subscales (median 2.2, SD 0.73; Table 2).

**Table 2.** *Technikaffinität–Elektronische Geräte* (Affinity for Technology–Electronic Gadgets) score of the sample (N=326).

Scales	Score, median (SD)
Enthusiasm for technology	2.2 (0.72863)
Competence in using technology	2.75 (0.43531)
Fear of technology	3 (0.43722)
Trust in technology	3.6 (0.562)
Overall score	2.9474 (0.3812)

### Use of Digital Media in General and in Everyday University Life

Digital media were most frequently used for communication (n=320, 98.2%), information search (n=291, 89.3%), and entertainment (n=268, 82.2%), while work-related activities occurred daily in approximately half of the sample (n=180, 55.2%). Video gaming and online shopping were less common (Multimedia Appendix 1).

Most students preferred desktop PCs, tablets, or smartphones as end devices for DGBL and expressed a positive intention to use DGBL in the future (agree: n=140, 42.9%; tend to agree: n=94, 28.8%). DGBL was perceived as particularly useful in modules such as Prevention and Health Promotion, Epidemiology and Statistics, and Population Medicine, with practical applications (including exam preparation, knowledge checks, and learning new content) rated most positively. Open-ended responses emphasized that DGBL should supplement, not replace, face-to-face teaching and support self-directed learning.

Gaming motivations were primarily entertainment, relaxation, and boredom, while competitive or stress-relief motives were less relevant. Digital media in everyday university life were rated as most important for internet access, PCs or tablets, and streaming services, while print media, computer games, and educational games were less relevant. Regarding the specific use of educational games, most participants stated that they did not use them (n=306, 93.9%). Only a small minority of students (n=20, 6.1%) reported using educational games, most frequently Duolingo (Duolingo Inc) and Babbel (Babbel AG), while other apps (eg, Atomas, BioApp, Cross Logic, Icare, Comma Rules, King of Mathematics, educational games for children, 10 Finger Training, Speexx [Speexx GmbH], Simulation Game, Studysmarter [StudySmarter GmbH], and other logic games) were used individually (<5% each; Multimedia Appendix 2).

### Attitudes of Students Toward the Use of DGBL in Public Health Study Programs and Health Sciences

In response to a DGBL-oriented learning game scenario (Multimedia Appendix 3), students predominantly preferred desktop PCs (n=119, 36.5%), tablets (n=100, 30.7%), or smartphones (n=91, 27.9%) as end devices, while virtual reality or augmented reality glasses and game consoles were rarely chosen (n = 6, <2%). Most participants reported a willingness to use DGBL in the future (agree: n=140, 42.9%; tend to agree: n=94, 28.8%), whereas 9.5% (n=31) disagreed and 18.7% (n=61) did not provide information.

### Perceived Potentials of DGBL in Public Health Study Programs and Health Sciences

Regarding the perceived usefulness of DGBL across modules (Multimedia Appendix 4), the highest levels of agreement were found for Prevention and Health Promotion (n=277, 85%), Epidemiology and Statistics (n=271, 83%), Population Medicine (n=251, 77%), and Environment and Health (n=271, 83%). Moderate agreement was observed for demography, health system, policy, or sociology, and health economics or management, whereas nursing sciences (n=104, 32%) and health services research (n=124, 38%) received lower levels of agreement. Open-ended responses highlighted additional applications in eHealth, communication strategy, scientific writing, and other specialized modules, with 1 participant emphasizing potential usefulness “in all modules of all subjects” (R12). Refer to Multimedia Appendix 5 for participants’ personal assessments of the use of DGBL (N=326).

Participants rated practical DGBL applications highly, especially exam preparation (n=279, 85.6%), knowledge checks (n=282, 86.5%), and acquiring new knowledge (n=273, 83.8%). Moderate support existed for methodological training (n=252, 77.3%), motivation to study (n=242, 74.2%), visualizing practical applications (n=242, 74.2%), error analysis, personalized learning profiles, self-determined action, and co-design (which received majority agreement but lower percentages; Multimedia Appendix 5).

Open-ended comments emphasized that DGBL should be used to supplement, rather than replace, face-to-face teaching. In addition, it should support self-study and be piloted in suitable modules to allow the accumulation of practical experience. Participants also emphasized that DGBL should be applied where it is didactically meaningful, supplementing rather than replacing face-to-face teaching and supporting low-threshold self-study, motivation, and structured learning. Some students suggested broader integration of serious games, while others noted that more experience is needed to assess DGBL’s potential, recommending piloting in suitable modules (R119, R146, R214, R250, and R292). The open-ended field also revealed that participants considered the use of DGBL to be useful in other modules, including e–Public Health or eHealth (n=3, 0.9%); communication strategy (n=2, 0.6%); and scientific writing (small paragraphs), strategic research, social research, nursing education, psychology, statistics, biomedicine, and counseling and interviewing (each n=1, 0.3%). One person also emphasized that DGBL would be useful “in all modules of all subjects” (R12).

In an open-ended question, the respondents were able to emphasize their key comments on potential uses of DGBL in public health teaching. In terms of possible applications, DGBL should be used specifically where it makes didactic sense:

*DGBL should only be used where it makes sense.*

[R119]

However, according to some participants, DGBL applications should not replace face-to-face teaching, but merely supplement it, especially for the voluntary consolidation of content or learning control (R264). In addition, DGBL applications can motivate and structure self-study phases in a low-threshold manner (R146, R214, and R317). Some students emphasized the desire for a fundamental use of DGBL in their studies:

*I would like to learn more through serious games.*

[R129 and R250]

Others highlight that the idea of DGBL is still too abstract and that more experience with DGBL needs to be gained to enable a well-founded assessment of possible applications (R184 and R292). This could be remedied by piloting different DGBL applications in suitable modules (R292).

### Promoting and Inhibiting Conditions for the Use of DGBL in Public Health Study Programs and Health Sciences

Students highlighted procedural and structural facilitators (Multimedia Appendix 6). Most agreed on the importance of data protection (n=303, 93%), technical infrastructure (n=321, 98.5%), and teaching staff skills (n=311, 95.4%). Adequate time allocation (n=310, 95.1%), central IT support (n=263, 80.7%), accessibility (n=303, 92.9%), end devices (n=247, 75.8%), and tutors (n=235, 72.1%) were also relevant. A majority agreed that prior skills may be necessary (n=183, 56.1%).

Additional conditions focused on usability and learning experience (Multimedia Appendix 7). Key requirements were ease of use (n=318, 97.5%), clarity (n=314, 96.3%), feedback (n=315, 96.6%), the ability to play anytime and anywhere (n=307, 94.2%), and technical reliability (n=299, 91.7%). A playful experience without time pressure (n=270, 82.8%), multimedia elements (n=235, 72.1%), reward systems (n=230, 70.5%), excitement (n=223, 68.4%), and realistic storylines (n=190, 58.3%) were also valued. In contrast, 3D graphics, immersion, avatar use, and multiplayer modes were less relevant. Nonuse was mainly reported because of concerns about additional sedentary PC activities (Multimedia Appendix 8).

Social influence (Multimedia Appendix 9) came primarily from fellow students (n=248, 76.2%) and lecturers (n=249, 76.6%), with lesser influence from university information sources, friends, partners, family, social media, and the press.

During gameplay, motivating factors included appropriate challenges (n=314, 96.3%), reflection on learning (n=297, 91.1%), feedback (n=300, 92%), individual performance measurement (n=242, 90.8%), and personal addressing of content (n=259, 79.3%). Social interaction (n=186, 57.1%), immersion in the history (n=197, 60.5%), realism of the story (n=180, 55.3%), team play, and competition were less influential (Multimedia Appendix 10).

In a final open-ended question, the respondents were able to emphasize their key comments on promoting and inhibiting conditions for the use of DGBL in public health teaching. The content and visual design of DGBL should be visually appealing, authentic, and serious without appearing childish (R87 and R184). In addition, clear feedback loops should be integrated so that players immediately recognize whether their answers are right or wrong and have a sense of participation and success (R164). From 1 participant’s own experience, the consideration of social aspects in video games was considered relevant but not decisive for learning success:

*Many of the video games that helped me on a personal level had no social aspect.*

[R162]

Regarding access to DGBL, “soft digitalization” is preferred, which refers to gradual infrastructural adjustments rather than abrupt implementation:

*Instead of hard digitalization = soft digitalization!*

[R111]

## Discussion

### Principal Results

Most students reported a positive attitude toward DGBL in public health and health sciences, with strong support for future implementation. DGBL was perceived as particularly useful for Prevention and Health Promotion, Epidemiology and Statistics, and for exam preparation and knowledge monitoring. Desktop PCs, tablets, and smartphones were all considered suitable devices.

Successful implementation was seen as contingent on reliable technical infrastructure, media-competent teaching staff, data protection, and high usability. Students emphasized flexibility, clarity, and a pressure-free learning environment. Although multimedia elements were generally welcomed, immersive features such as 3D graphics or multiplayer formats were considered less relevant.

The technology affinity profile revealed moderate competence and trust but low enthusiasm and high technology-related fear, indicating cautious engagement with DGBL. Accordingly, students favored structured, predictable, and functionally transparent designs. Peer and instructor support were identified as key factors for acceptance.

Overall, students expressed openness to DGBL when embedded in clear, supportive, and goal-oriented learning contexts that emphasize personal progress, feedback, and emotional safety over competitive or immersive mechanisms.

### Comparison With Prior Work

Previous literature has broadly demonstrated the educational potential of serious games in health care training. Chang et al [23] have highlighted that DGBL can significantly improve learning performance, self-efficacy, and satisfaction among nursing students. Similarly, Peng [22] showed that educational games in health communication settings could effectively promote healthier dietary behaviors. However, these examples predominantly focus on applied medical settings or individual behavior change and often target medical or nursing students rather than the broader interdisciplinary scope of public health education. Systematic reviews [24-27] support the claim that serious games can enhance learning enjoyment, knowledge retention, and skill acquisition across multiple health disciplines. Nevertheless, the scope of these reviews primarily includes physiotherapy, psychology, or general medical education. The present study addresses this oversight by concentrating on public health students, a population largely neglected in the literature. Moreover, although prior studies [19-21] have examined the use of serious games in various academic contexts, few have focused on students’ attitudinal and contextual prerequisites for adopting DGBL. Our study contributes by empirically documenting both the facilitators and barriers to DGBL integration, including infrastructural needs, pedagogical considerations, and motivational dynamics, such as feedback and challenge. In contrast to previous works, we offer a user-centered view grounded in the actual preferences, reservations, and expectations of students enrolled in public health–related programs. Rather than evaluating a specific DGBL application, as seen in most intervention-based designs [18], our research used a scenario-based survey to explore students’ anticipated acceptance and perceived utility of DGBL across a variety of modules and use cases. This broader approach allows a more generalizable understanding of DGBL’s potential role in public health curricula. Finally, although the *Hochschulforum Digitalisierung* [3] and other German initiatives have promoted digital innovation in higher education, their recommendations often remain theoretical or focus on conventional digital tools such as e-learning platforms and lecture recordings. In contrast, our study assesses students’ receptiveness to more immersive, interactive forms of digital education such as serious games and identifies practical implementation factors such as device preferences, flexibility in timing, and game design expectations. In sum, we complement and extend prior research by focusing on a previously underexplored academic population—public health students—and by detailing both their general attitudes toward DGBL and the specific structural, pedagogical, and social factors shaping its potential uptake in university-level health education.

### Limitations

One limitation of this study is the occasional technical issue affecting the display of the DGBL scenario within the survey. At least 2 participants reported that the example scenario was not rendered correctly when using Safari on a MacBook. This may have affected their understanding of the scenario and increased the abstraction level of the subsequent questions, potentially influencing their responses. Future studies should ensure compatibility across different browsers and devices to improve data quality and user experience. In addition, usability testing should be conducted prior to data collection to identify and minimize such issues.

Another limitation concerns the sample composition and recruitment. Although students from multiple universities and health-related programs were included, recruitment via social media and voluntary participation may have led to self-selection bias, as students with greater interest in digital learning were likely more inclined to participate. Due to the anonymous online format, it was theoretically possible for students to complete the questionnaire multiple times. However, as invitations were primarily sent to new students each semester, the likelihood of multiple participation was minimized. Because the total number of students reached is unknown, a traditional response rate could not be calculated. Consequently, the sample represents only a subset of the student population in Germany and cannot be generalized to all public health and health sciences students.

The extended data collection period from 2021 to 2024 also presents a limitation, as it spans different phases of the COVID-19 pandemic and the postpandemic period. Students’ experiences and familiarity with online learning may have changed during this time, potentially influencing attitudes toward DGBL. Temporal changes were not analyzed in this exploratory study. Future research could examine cohort or time effects to account for such variation.

Finally, the analysis was limited to descriptive statistics and qualitative content analysis. Although this approach allowed an exploratory understanding of student attitudes and conditions for DGBL use, causal conclusions or inferential generalizations are not possible. Future studies could apply mixed methods approaches or longitudinal study designs to assess effectiveness, behavioral outcomes, and changes in attitudes over time.

### Conclusions

Our study addresses an underexplored area by examining DGBL in public health and health sciences education in Germany. The findings indicate an overall positive attitude among students toward DGBL, particularly for practice-oriented applications such as exam preparation, knowledge testing, and the visualization of complex content. Students identified Prevention and Health Promotion, Epidemiology and Statistics, and Population Medicine as especially suitable domains. Effective implementation of DGBL requires adequate technical infrastructure, media-competent teaching staff, and a strong focus on usability, data protection, and pedagogical integration. Given students’ moderate technological competence but high technology-related fear and low enthusiasm, DGBL designs should prioritize clarity, reliability, and psychological accessibility over immersive or complex features. Academic peers and instructors play a key role in shaping acceptance. Future research should assess longitudinal learning effects and implementation feasibility across institutional contexts to support the sustainable integration of DGBL into public health and health sciences curricula.

## Supplementary material

10.2196/83347 Multimedia Appendix 1 Use of digital media (N=326).

10.2196/83347 Multimedia Appendix 2 Reasons for playing computer or video games (N=326).

10.2196/83347 Multimedia Appendix 3 Example scenario.

10.2196/83347 Multimedia Appendix 4 Assessment of the usefulness of digital game–based learning in public health–related modules (N=326).

10.2196/83347 Multimedia Appendix 5 Personal assessment of the use of digital game–based learning (N=326).

10.2196/83347 Multimedia Appendix 6 Assessment of the framework conditions for the use of digital game–based learning at the university (N=326).

10.2196/83347 Multimedia Appendix 7 Assessment of the framework conditions for the use of digital game–based learning in health-related programs (N=326).

10.2196/83347 Multimedia Appendix 8 Personal assessment of the nonuse of digital game–based learning (N=326).

10.2196/83347 Multimedia Appendix 9 Assessment of social influence on the use of digital game–based learning at the university (N=326).

10.2196/83347 Multimedia Appendix 10 Assessment of motivation during the game (N=326).

## Abbreviations

ASPHER: Association of Schools of Public Health in the European Region
DGBL: digital game–based learning
TA-EG: *Technikaffinität–Elektronische Geräte*
WHO: World Health Organization
